# Prognostic Value of Malondialdehyde (MDA) in the Temporal Progression of Chronic Spinal Cord Injury

**DOI:** 10.3390/jpm13040626

**Published:** 2023-04-02

**Authors:** Sergio Haro Girón, Jorge Monserrat Sanz, Miguel A. Ortega, Cielo Garcia-Montero, Oscar Fraile-Martínez, Ana M. Gómez-Lahoz, Diego Liviu Boaru, Diego de Leon-Oliva, Luis G. Guijarro, Mar Atienza-Perez, David Diaz, Elisa Lopez-Dolado, Melchor Álvarez-Mon

**Affiliations:** 1 Department of Medicine and Medical Specialities, Faculty of Medicine and Health Sciences, University of Alcalá, 28801 Alcalá de Henares, Spain; 2 Ramón y Cajal Institute of Sanitary Research (IRYCIS), 28034 Madrid, Spain; 3 Unit of Biochemistry and Molecular Biology, Department of System Biology (CIBEREHD), University of Alcalá, 28801 Alcala de Henares, Spain; 4 Service of Rehabilitation, National Hospital for Paraplegic Patients, Carr. de la Peraleda, S/N, 45004 Toledo, Spain; 5 Service of Internal Medicine and Immune System Diseases-Rheumatology, University Hospital Príncipe de Asturias, (CIBEREHD), 28806 Alcalá de Henares, Spain

**Keywords:** malondialdehyde (MDA), spinal cord injury (SCI), plasma biomarker, oxidative stress, lipid peroxidation, prognosis

## Abstract

Background: Oxidative stress is a major signature of spinal cord injury (SCI). The altered levels of various oxidative stress markers have been demonstrated in acute and chronic SCI. However, the variation of these markers in patients with chronic SCI depending on the time since the initial injury has not been explored yet. Objective: Our aim was to measure plasma levels of malondialdehyde (MDA), a marker of lipid peroxidation in patients with SCI stratified in different periods of suffering the injury (0–5 years, 5–10 years, and more than 10 years). Patients and methods: This cross-sectional study enrolled patients with SCI (N = 105) from different periods of the lesion and healthy control (HC) subjects (N = 38): short period (SCI SP, N = 31, time of evolution less than 5 years); early chronic (SCI ECP, N = 32, time of evolution 5–15 years); and late chronic (SCI LCP, N = 42, time of evolution more than 15 years). The plasma levels of MDA were measured using a commercially available colorimetric assay. Results: Patients with SCI had significantly higher plasma levels of MDA than HC subjects. Receiver operating characteristic (ROC) curve analysis for plasma MDA levels in patients with SCI demonstrated areas under the curve (AUC) of 1 (HC vs. SCI-SP); 0.998 (HC vs. SCI-ECP); and 0.964 (HC vs. SCI-LCP). Additionally, three ROC curves were used to compare the different concentrations of MDA between the subgroups of patients with SCI, and the resulting AUCs were: 0.896 (SCI-SP vs. SCI-ECP); 0.840 (SCI-ECP vs. SCI-LCP); and 0.979 (SCI-SP vs. SCI-LCP). Conclusion: Plasma concentration of MDA can be considered as an oxidative stress biomarker to assess the prognosis of SCI in chronic stages.

## 1. Introduction

Spinal cord injury (SCI) is a disabling neurological condition that disrupts major motor, sensory, and autonomic functions [1]. Patients suffering from SCI course with serious multisystem comorbidities need intensive care unit management [2]. Although epidemiological data are quite sparse, recent systematic reviews have found that there are nearly 500 cases of SCI per million people in developed countries and 440 per million in developing ones [3]. The incidence of traumatic SCI seems to keep increasing, especially in youth from developed areas of the U.S. and Europe. The leading causes are preventable vehicle accidents and falls [4]. Although the acute care of SCI has significantly improved in recent years with a reduction in mortality, the clinical management of patients with chronic SCI still represents a notable medical challenge to face [5]. 

Initial trauma is irreversible and is called primary injury. This phase comprises mechanical forces due to displaced bone fragments, disc materials, and/or ligaments bruising or tearing into the spinal cord tissue [6]. Consequently, loss of blood vessels and neuronal and glial cells after primary injury drives environmental changes in the spinal cord, designated as secondary injury [7]. There are many pathophysiological mechanisms involved in the secondary injury, including neurogenic shock, vascular insults (i.e., hemorrhage and ischemia-reperfusion phenomena), excitotoxicity, calcium-mediated secondary injury, enhanced cell death, and so on [8]. The secondary injury occurs in three main phases: acute (minutes to hours), sub-acute (hours to months), and chronic (months to years). Although the transition between different phases cannot be specifically predicted, the chronic phase is considered the largest period of the patient´s life, presenting a broad spectrum of local and systemic alterations that remains to be fully understood [9]. 

In this sense, the role of local and systemic inflammation (measured by cytokines and cellular subsets) and oxidative stress has been contemplated in previous works, representing a puzzle piece to understanding the progression from acute/subacute to chronic stages [10,11]. Oxidative stress occurs when reactive oxygen species (ROS) are accumulated beyond the body’s antioxidant capacity. Even during normal metabolic processes, ROS are produced as a byproduct, but in the event of an SCI, ROS production becomes exacerbated, causing an imbalance and cells’ inability to detoxify. The role of oxidative stress is complex in SCI pathogenesis and involves multiple mechanisms [12]. 

The stimulation of inflammatory pathways is one of the main mechanisms of oxidative stress in SCI. Immune cells such as macrophages and microglia are activated after damage and release cytokines and chemokines that promote inflammation [13,14]. These compounds have the potential to produce ROS, which in turn has the potential to activate further inflammatory pathways. This vicious cycle of inflammation and oxidative stress can worsen tissue damage and hasten the onset of chronic SCI.

Another mechanism of oxidative stress in SCI is related to mitochondrial dysfunction. Following SCI, mitochondria can become damaged, leading to a decrease in energy production—which is supposed to be the main function of this organelle—and an increase in ROS production [15,16]. This can further aggravate oxidative stress and contribute to tissue damage, with characteristic clinical manifestations in this pathology, such as pressure ulcer formation [17].

Oxidative stress may also have an impact on cell signaling pathways that are essential for cell survival and repair. The NF-kB pathway, for example, is activated by oxidative stress and regulates the expression of genes involved in inflammation and cell survival [18]. Persistent activation of this system, however, may harm tissues and cause cell death. Neurological damage and neuron loss can provoke oxidative stress [19]. When neurons are damaged or die, they release ROS and other toxic molecules that can lead to oxidative stress [14,20]. This can cause further damage to neighboring cells and exacerbate the initial injury or disease. In SCI, oxidative stress promotes further damage to neurons and accelerates their degeneration [21].

Overall, oxidative stress plays a significant role in the pathogenesis of SCI by contributing to inflammation, mitochondrial dysfunction, cell signaling dysregulation, and neuron loss. Therefore, targeting oxidative stress markers may have a promising value for diagnosis, prognosis, and therapeutic approaches for SCI.

There is also evidence to suggest that there is increased lipid peroxidation in neurons following spinal cord injury (SCI). Lipid peroxidation is a process in which free radicals attack and oxidize polyunsaturated fatty acids in cell membranes including neurons, leading to the generation of highly reactive products such as malondialdehyde (MDA) [22]. This increase in lipid peroxidation products can cause damage to the cellular membrane, leading to changes in membrane permeability and ion homeostasis and ultimately neurodegeneration and cell death [23].

In this work, we aim to focus on MDA, which is one of the main products of membrane lipid peroxidation, derived from polyunsaturated fatty acids (PUFAs) degradation. MDA is a highly reactive and toxic byproduct of lipid peroxidation [24]. MDA is considered a marker of oxidative stress because its accumulation is indicative of increased lipid peroxidation and ROS production [25]. Lipid peroxidation can also contribute to the progression of secondary injury mechanisms in the spinal cord, including inflammation and excitotoxicity [26]. Therefore, reducing lipid peroxidation may be a promising therapeutic target for the treatment of SCI.

MDA can react with cellular components such as proteins, DNA, and lipids, leading to cellular damage and dysfunction. Therefore, the presence of MDA in cells and tissues is often used as an indicator of oxidative stress, as it reflects the degree of lipid peroxidation and ROS-mediated damage.

This molecule has been suggested as a valuable biomarker with differences in physiological and pathological situations [27]. Measurement of MDA levels is widely used as a biomarker of oxidative stress and lipid peroxidation in clinical and experimental settings [28]. In addition, MDA can also be used as a therapeutic target for mitigating the harmful effects of OS, as antioxidants and other agents that reduce ROS production or scavenge free radicals can reduce MDA levels and attenuate oxidative stress [29].

MDA’s observation and application for assessing the acute phase of SCI have been explored, showing increasing levels from the first hours to the following days, but less is known about its abundance in chronic stages [30,31]. Research has shown that MDA plasma levels are significantly elevated in patients with acute SCI compared to healthy controls (HC). The increase in MDA levels is thought to be due to the generation of ROS as well as subsequent lipid peroxidation in the injured spinal cord tissue [32]. The high levels of MDA in the plasma of SCI patients may contribute to the secondary damage that occurs after the initial injury, including inflammation, apoptosis, and demyelination [33]. Furthermore, MDA has been shown to induce oxidative-stress-mediated cell death in various cell types including neurons and glial cells [34].

Therefore, the objective of the present work is to demonstrate if MDA could be a possible prognosis biomarker for chronic SCI patients to check the level of oxidative stress or lipid peroxidation in different periods of this stage (0–5 years, 5–10 years, and more than 10 years suffering the injury) compared to HC.

## 2. Methods

### 2.1. Patients Selection and the Study Protocol

We performed a prospective study on patients with chronic SCI (N = 105). To properly study the course of SCI, patients were stratified into three subgroups: short period (SCI SP, time of evolution less than 5 years); early chronic (SCI ECP, time of evolution 5–15 years); and late chronic (SCI LCP, time of evolution more than 15 years). SCI subjects were compared with sex- and age-matched HC (N = 38).

All subjects gave their signed informed consent, which had already been authorized by the National Hospital for Paraplegic Patients’ institutional review board (10 September 2015). The current study was performed under the fundamental ethical principles of autonomy, beneficence, non-maleficence, and distributive justice and by the statements of good clinical practice, the tenets of the most recent Helsinki Declaration (2013), and the Oviedo Convention (1997). The data and information were gathered following current data protection laws including Regulation (EU) 2016/679 and Organic Law 3/2018 of 5 December, which protects personal data.

Medical information from the SCI patients was collected in a routine clinical examination in the Physical Medicine and Rehabilitation Department, including the following data: (1) baseline demographic features; (2) time and mechanism of injury; (3) neurologic injury level and related severity; (4) tonic and phasic spasticity; (5) presence or absence of pain, its type, and its seriousness; (6) history of past infections and other indicators of a chronic SCI complication; (7) comorbidities; (8) use of contemporary medications; (9) fatigue; (10) anxiety and depression levels; (11) degree of independence in daily living activities; and (12) self-reported quality of life and health status. 

Blood samples were extracted from all individuals via standard venipuncture by an established aseptic technique. Samples were obtained from chronic SCI patients at the time of the medical evaluation in the outpatient clinic area.

The inclusion criteria considered in this study were the following: (1) being ≥18 years; (2) history of SCI in a period ≥1 year, occurring at any level; and (3) having SCI with varied severity, ranging from grades A to E according to the American Spinal Injury Association (ASIA) Impairment Scale (AIS) [35]. A Physical and Rehabilitation Medicine board-certified clinician in SCI medicine evaluated the subjects’ injuries according to the International Standards for Neurologic Classification of Spinal Cord Injury [36,37]. Conversely, exclusion criteria were as follows: (1) a coincident infection with notable severity, such as urinary tract infection (UTI) or a respiratory infection, evidenced with a positive culture in the last 3 months; (2) chronic viral or bacterial infection; (3) clinical diagnosis of an autoimmune disease; (4) serious cardiovascular disease (CVD); (5) hematopoietic, renal, lung, or hepatic complications; (6) an endocrine or metabolic disorder, (i.e., type 1/2 diabetes mellitus); (7) previous history of cancer; (8) pressure ulcers in the last year; (9) administration of immunomodulatory drugs such as steroids in the last 3 months; (10) suffering from immunodeficiency or malnutrition; (11) being in a pregnancy or lactation period; and (12) having undergone diagnosis of any psychiatric disorder.

### 2.2. Demographic Characteristics of the Cohort

The cohort of the study consists of 101 patients (mean age 35.23 ± 12.84% years, with 70.10% men) with chronic SCI and 40 HC (32.74 ± 8.92 years, with 63.70% men). The mean time of SCI onset was 12.99 ± 9.16.

The neurological level of spinal damage was located within C1–C4, C5–C8, T1–T6, and T7–T12 and the lumbosacral metamers in 23.8%, 20%, 26.27%, 20.95%, and 8.57% of patients, respectively. In other words, more than 70.40% of our patients had an SCI above T6. Concerning the ASIA, 46.67% of the patients were AIS A, 16.19% of the patients were AIS B, 16.19% of the patients were AIS C, and 20.95% of the patients were AIS D, indicating that although 79.04% of the patients exhibit incomplete lesions, just 62.85% reported incomplete motor injuries, with different extent of intralesional motor preservation and theoretically better mobility profiles. Overall, the demographic data of our chronic SCI patients and HC are collected in Table 1.

### 2.3. MDA Quantification

All subjects’ venipuncture was obtained using a recognized aseptic technique to acquire peripheral blood samples (BD Vacutainer^®^ with Lithium Heparin 68 IU). At the time of the evaluation, samples were collected and transferred to tubes of 15 mL. The blood samples were collected and centrifuged for 15 min at 15,000 rpm, and the plasma was separated from cellular content, aliquoted, and kept at −80 °C for additional examination.

Plasma MDA levels were determined using a colorimetric lipid peroxidation assay kit (ab118970; Abcam^®^, Cambridge, UK) following the manufacturer’s instructions. Thiobarbituric acid (TBA) and the MDA contained in the plasma sample (10 L) reacted to form an MDA-TBA adduct that could be measured colorimetrically. This test can find MDA at concentrations as low as 0.1 mol/well. A 0.1 mol/L MDA standard was created for the colorimetric test, and successive dilutions were made for the standard curve. An optical density of 532 nm was used to measure the MDA-TBA conjugate produced by the kit right away in a microplate reader (Victor 2 multipurpose device; Wallac, Victoria, Australia).

### 2.4. Statistics

GraphPad Prism 5.1 was used to conduct all statistical analyses (Graphpad Software Inc., San Diego, CA, USA). The data are displayed as mean SD or n of patients (%). The Mann–Whitney U-test was used to compare the data. The receiver operating characteristic (ROC) curves were analyzed for MDA. Plasma MDA data are displayed as a median and interquartile range (IQR). Statistical significance was defined as a *p*-value of 0.05.

## 3. Results

**Figure 1:**
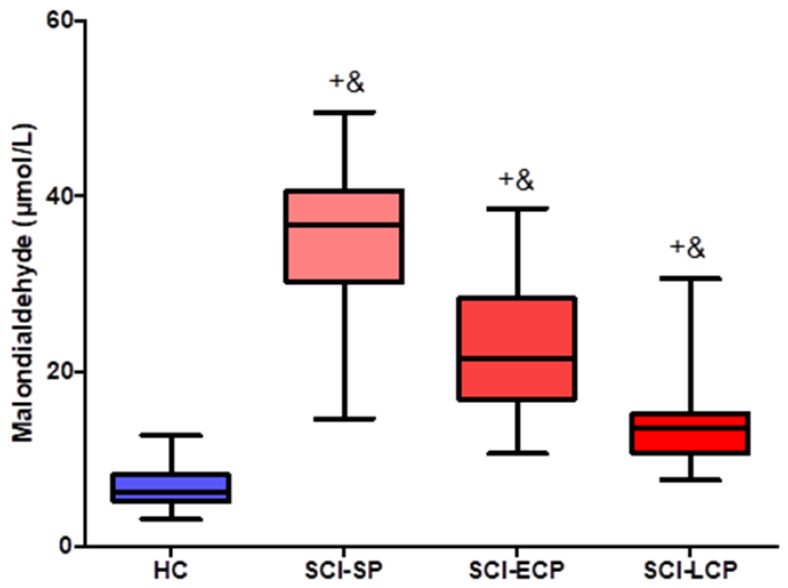
Boxplots of MDA concentrations according to healthy controls and different groups of patients with spinal cord injury. Data for plasma MDA levels are presented as median (horizontal black line inside) with interquartile range (IQR). HC, healthy controls (N = 38); SCI SP, short period (time of evolution less than 5 years) (N = 31); SCI ECP, early chronic (time of evolution 5–15 years) (N = 32); SCI LCP, late chronic (time of evolution more than 15 years) (N = 42). HC vs. SCI-SP = + = *** *p*  < 0.0001; HC vs. SCI-ECP = + = *** *p* < 0.0001; HC vs. SCI-LCP = + = *** *p* < 0.0001; SCI-SP vs. SCI-ECP = & = *** *p* < 0.0001; SCI-ECP vs. SCI-LCP = & = *** *p* < 0.0001; SCI-SP vs. SCI-LCP = & = *** *p* < 0.0001; Mann–Whitney U-tests.

We extrapolated plasma levels of MDA in patients with SCI at different stages from the colorimetric analysis. The three subgroups of patients showed significantly more increased levels of MDA than HC (*p <* 0.0001), and significant differences among the groups were also found, showing a decreased tendency of MDA concentrations along the different stages of the disease (*p <* 0.0001). The median (IQR) plasma MDA levels were HC = 6.142 (3.125–12.652) µmol/L; SCI SP = 36.655 (14.585–49.569) µmol/L; SCI ECP = 21.449 (10.564–38.516) µmol/L; SCI LCP = 13.601 (7.568–30.561) µmol/L. Comparisons can be observed in detail in Figure 1.

**Figure 2:**
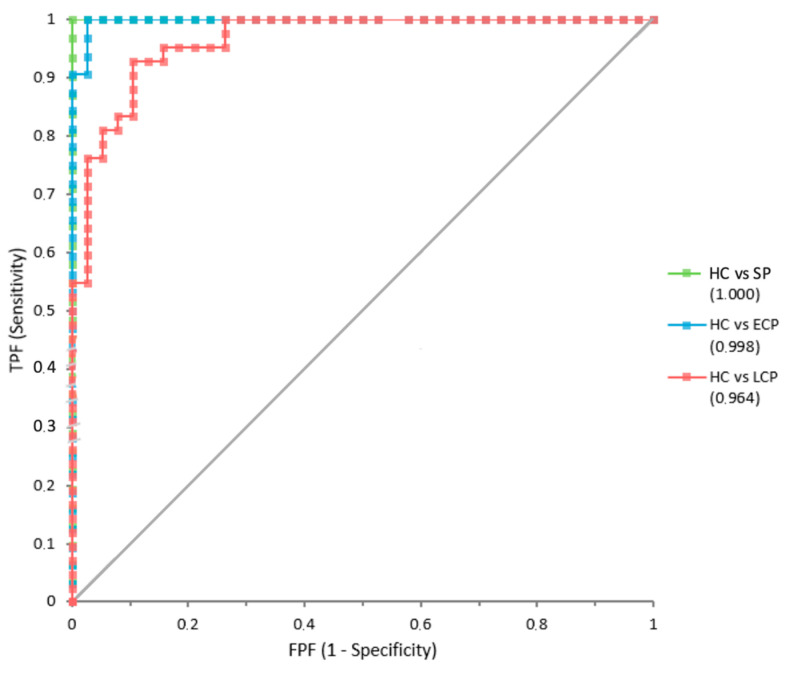
ROC curves for MDA comparing subgroups of patients with SCI to HC. TPF, true positive false (sensitivity); FPF, false positive false (1-specificity). AUC for HC vs. SP was 1 (green), HC vs. SCI-ECP was 0.998 (blue), and HC vs. SCI-LCP was 0.964 (red).

We performed several ROC curve analyses. On one hand, ROC curves analyses for plasma MDA levels comparing HC and patients with SCI were represented with three curves, one per group, which can be observed in Figure 2. SCI-SP showed an area under the curve (AUC) of 1 (±SE = 0), SCI-ECP patients showed an AUC of 0.998 (±0.0028), and SCI-LCP an AUC of 0.964 (±0.0176). 

**Figure 3:**
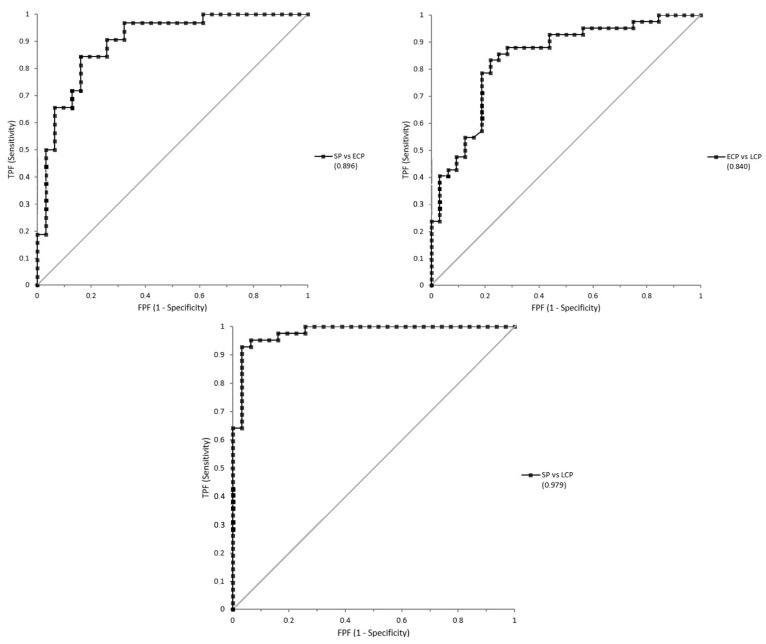
ROC curves for MDA concentrations comparing three subgroups of patients with SCI. SCI-SP vs. SCI-ECP curve presents an AUC of 0.896; SCI-ECP vs. SCI-LCP curve presents an AUC of 0.840; and SCI-SP vs. SCI-LCP presents an AUC of 0.979.

On the other hand, three ROC curves were additionally used to compare the different concentrations of MDA between the subgroups of patients with SCI. the SCI-SP vs. SCI-ECP curve showed an AUC of 0.896 (±0.0401); the SCI-ECP vs. SCI-LCP curve showed an AUC of 0.840 (±0.0473); and finally, the SCI-SP vs. SCI-LCP curve showed an AUC of 0.979 (±0.0138), all of which are represented in Figure 3.

## 4. Discussion

The present study demonstrated significantly higher plasma levels of MDA in patients with SCI. The longer patients continued with the lesion, the more concentrations of MDA tended to decrease. MDA concentrations seem to stabilize over time among SCI subgroups but are, in any case, higher than in the HC group. Even in the SCI-LCP group, concentrations are higher than in HC. These data show information about the status of membrane lipid peroxidation in patients with SCI, with MDA being an easily measurable marker in plasma from these patients.

Oxidative stress is considered a hallmark of SCI. After the primary injury, secondary mechanisms of damage trigger oxidative damage, which in turn exacerbates further injury [11]. Oxidative stress can be defined as “the consequence of the loss of the balance between oxidants and antioxidants in favor of the former” [38]. A massive oxidant production (mainly represented by reactive oxygen species (ROS) and reactive nitrogen species (RNS)) and a decreased concentration of antioxidants can be observed after SCI, promoting serious damage to proteins, lipids, and DNA [39]. In more detail, proteins and DNA damage favors direct initiation of necrosis and programmed cell death, whereas lipid peroxidation seems to be involved in membrane dysfunction, inhibition of calcium pump and Na^+^/K^+^ ATPase, and calcium accumulation, eventually resulting in cell death as well [39,40]. The relevance of oxidative stress has been demonstrated in both acute and chronic phases. Specifically, it is known that oxidative stress is tightly linked to the immune dysfunction associated with SCI, both being factors responsible in combination for several mechanisms observed in acute and chronic SCI patients [41,42]. 

Previous studies have deeply explored oxidative stress routes in SCI and have aimed to find new biomarkers as therapeutic targets. MDA represents an attractive marker of oxidative stress and lipid peroxidation, having been demonstrated as an important upregulation in acute stages of SCI [31,43,44]. At chronic stages, previous works have identified that patients with chronic SCI tend to have altered levels of MDA and other oxidative stress markers when compared to healthy subjects [45]. However, to our best knowledge, this is the first study evaluating the levels of MDA in patients with chronic SCI considering the period of evolution. It is widely accepted that there are significant functional changes over time in chronic SCI [46]. Despite secondary mechanisms of damage that tend to stabilize after acute and subacute stages, there is evidence that patients with chronic SCI present a broad spectrum of systemic alterations, making them more prone to suffer from various complications and morbidities [47,48,49]. Although there is little information available about how oxidative stress varies after the temporal progression of SCI, our results seem to indicate that patients in the subchronic phase (1 to 5 years) had an evident increase of lipid peroxidation when compared to those in early chronic phase (between 5 and 15 years), and this was even more evident when compared to those in late chronic phase (more than 15 years). 

Although the groups of patients are significantly representative, this study has limitations. Some of them reside in not considering the age of patients, as the body’s antioxidant capacity decreases with aging, leading to a greater accumulation of ROS and increased oxidative stress [50,51] as well as boosting microglial activation [52]. Moreover, it is of note to consider emotional stress as a source of ROS as well now that cortisol leads to their production [53,54,55]. Thus, there could be interindividual differences in this sense, linking their psychological stress with oxidative stress levels and then MDA levels.

Lipid peroxidation plays an important role in the pathophysiology of SCI and can lead to damage to neurons and other cells in the spinal cord [32]. All in all, the fact that MDA values were higher than the HC in all chronic SCI patients seems to support that there is persistent oxidative stress and lipid peroxidation observed in these subjects, although the sources of free radicals and the alterations in the antioxidant levels can be different in each phase. In this sense, and after elaborating an ROC curve, we showed that MDA can be considered a valuable prognostic value to differ lipid peroxidation levels across the distinct phases of chronic SCI and with healthy subjects. This knowledge can support the relevance of addressing oxidative stress in the early stages of chronic SCI, particularly by considering antioxidants, nutritional interventions, and different bioactive compounds/nutraceuticals (i.e., polyphenols such as curcumin or quercetin), which could entail potential benefits in these patients [49,56,57]. Additionally, certain clinical trials are working toward this goal. Methylprednisolone, a glucocorticoid steroid, and tirilazad, a non-glucocorticoid 21-aminosteroid, have both been demonstrated in clinical trials to have strong antioxidant properties and to aid in the recovery of SCI patients. The molecular targets and methods of action of additional interesting plant chemicals with the potential to prevent SCI have also been revealed. Carotenoids and phenolic chemicals are some of these [11]. Reducing exposure to these sources of oxidative stress and guaranteeing healthy lifestyle habits including regular physiotherapy and a balanced diet can help minimize the impact of oxidative stress.

## 5. Conclusions

In the present work, we demonstrate a differential detection of plasma levels of MDA, a marker of lipid peroxidation, in patients with chronic SCI according to the period of evolution. In more detail, we show that patients at subchronic stages (1 to 5 years post injury) present the highest levels of this marker, whereas levels of MDA tend to decrease and stabilize over time. Nevertheless, MDA levels remain consistently elevated over time in comparison with HC subjects. 

Monitoring MDA plasma levels in SCI patients may provide valuable information on the extent of oxidative stress and lipid peroxidation in the injured spinal cord tissue. Additionally, strategies aimed at reducing oxidative stress and lipid peroxidation may be potential therapeutic targets for the treatment of SCI.

Our ROC analyses further support the prognostic value of this marker in chronic stages, although future studies should be aimed at evaluating possible differences in oxidative stress markers and mechanisms across chronic SCI patients along with possible strategies directed to ameliorate oxidative damage in this population.

## Figures and Tables

**Table 1:** Demographic data of healthy controls (HC) and SCI patients. SCI with short period of evolution (SCI SP), if the time since initial injury was comprised between 1 to 5 years; chronic SCI at early chronic phase (SCI ECP) if the time of evolution was between 5 and 15 years; and chronic SCI at late chronic phase (SCI LCP) if the time of evolution was more than 15 years. ASIA = American Spinal Injury Association.

Variable	HC (N = 38)	SCI (N = 105)	SCI SP (N = 31)	SCI ECP (N = 32)	SCI LCP (N = 42)
Age (years)	25.41 ± 7.99	36.33 ± 13.24	29.24 ± 14.40	36.81 ± 12.26	40.28 ± 17.65
Gender (men/women)	42.10%/57.90%	74.28%/25.72%	90.32%/9.68%	77.78%/22.22%	61.70%/38.30%
Time of injury (years)	__	13.24 ± 9.47	2.30 ± 1.54	10.11 ± 2.55	22.26 ± 5.33
ASIA					
A		46.67%	41.93%	55.55%	44.68%
B		16.19%	3.70%	3.70%	21.28%
C		16.19%	18.51%	18.52%	19.15%
D		20.95%	22.22%	22.22%	14.89%
Injury level					
C1–C4		23.80%	22.22%	22.22%	14.89%
C5–C8		20.00%	18.51%	18.52%	25.53%
T1–T6		26.27%	25.92%	25.93%	29.79%
T7–T12		20.95%	29.62%	29.63%	14.89%
L1–L6		8.57%	3.70%	3.70%	14.89%

## Data Availability

The data used to support the findings of the present study are available from the corresponding author upon request.
